# Different effects of Wnt/β-catenin activation and PTH activation in adult and aged male mice metaphyseal fracture healing

**DOI:** 10.1186/s12891-020-3138-3

**Published:** 2020-02-19

**Authors:** Daocheng Liu, Hao Qin, Jiazhi Yang, Lei Yang, Sihao He, Sixu Chen, Quanwei Bao, Yufeng Zhao, Zhaowen Zong

**Affiliations:** State Key Laboratory of Trauma, Burn and Combined Injury, Department of War Wound Rescue Skills Training, Base of Army Health Service Training, Army Medical University, Chongqing, 400042 China

**Keywords:** Wnt/β-catenin, PTH, Fracture healing, Adult, Aged

## Abstract

**Background:**

Fractures in older men are not uncommon and need to be healed as soon as possible to avoid related complications. Anti-osteoporotic drugs targeting Wnt/β-catenin and PTH (parathyroid hormone) to promote fracture healing have become an important direction in recent years. The study is to observe whether there is a difference in adult and aged situations by activating two signal paths.

**Methods:**

A single cortical hole with a diameter of 0.6 mm was made in the femoral metaphysis of Catnb^lox(ex3)^ mice and wild-type mice. The fracture healing effects of CA (Wnt/β-catenin activation) and PTH (activated by PTH (1–34) injections) were assessed by X-ray and CT imaging on days 7, 14, and 21 after fracture. The mRNA levels of β-catenin, PTH1R(Parathyroid hormone 1 receptor), and RUNX2(Runt-related transcription factor 2) in the fracture defect area were detected using RT-PCR. Angiogenesis and osteoblasts were observed by immunohistochemistry and osteoclasts were observed by TRAP (Tartrate-resistant Acid Phosphatase).

**Result:**

Adult CA mice and adult PTH mice showed slightly better fracture healing than adult wild-type (WT) mice, but there was no statistical difference. Aged CA mice showed better promotion of angiogenesis and osteoblasts and better fracture healing than aged PTH mice.

**Conclusion:**

The application of Wnt/β-catenin signaling pathway drugs for fracture healing in elderly patients may bring better early effects than PTH signaling pathway drugs, but the long-term effects need to be observed.

## Background

Over 100,000 fractures are sustained every year in the UK by people of all ages and sexes [1]. Nonunion is an important complication of fractures. The most common risk factors include diabetes, increased age, osteoporosis, excessive alcohol consumption, blood circulation, and non-steroidal anti-inflammatory drugs (NSAIDs) [2]. Metaphyseal fractures are a major occurrence; hip fractures account for 3.5% of fractures in older women and 2.6% in older men [3]. More attention has been given to estrogen-related osteoporotic fractures in older women, while osteoporotic fractures in older men have been studied less frequently [4, 5]. Decreased bone mineral density may lead to osteoporotic fractures, which occur frequently in both older men and women. Male predisposing factors include reduced body mass index, family history of osteoporosis, and increased age [6, 7].

The mechanisms underlying fracture healing are of utmost importance for delineating the possible causes of impaired bone repair, as well as for identifying pharmaceutical agents that could be used to accelerate the healing process and increase bone union rates. Physiological bone repair involves a complex series of events.

In recent years, Wnt/β-catenin activation and PTH (parathyroid hormone) have been studied for the treatment of osteoporosis and the promotion of fracture healing. The classical Wnt/β-catenin signaling pathway plays an important role in bone regeneration and remodeling in mice and humans. While activation of the Wnt/β-catenin signaling pathway cannot prevent or treat osteoporosis, it can promote fracture healing [8–10]. PTH has been widely used clinically in the treatment of osteoporosis. Recently, some studies have found that intermittent low-dose parathyroid hormone can promote fracture healing in animals and humans and firm the fixation of the implants [11–17]. Although the two signaling pathway are known to have a role in promoting fracture healing, it is unclear whether their effects are the same at different ages of the patient. Observing the effects of activating two signaling pathways that promote fracture healing in both adult and elderly patients will provide some guidance for the clinical selection of drugs for patients of different ages, especially in the early stages of fracture. In particular, promoting fracture healing is an urgent need for elderly patients.

## Methods

### Animals

All animal procedures were approved by the Laboratory Animal Welfare and Ethics Committee of the Third Military Medical University of China and were conducted in accordance with procedures approved by the institution. The TM-inducible Cre fusion protein Cre-ERTM expression is under the control of a 3.2 kb mouse procollagen 1 promoter (3.2 kb Col1-Cre). ERTM crossed with Catnb+/flox (exon 3) mice (obtained from Feng Jianquan, Department of Biomedical Sciences, Baylor Dental University, USA) [15], and genotype mice without expressed in littermates. The 3.2 kb Col1-Cre ERTM/β-catenin exon 3 fx+/+ activates the β-catenin signaling pathway in mouse osteoblasts by TM injection [18, 19]. Genotyping was performed with a routine method. Briefly, DNA was extracted from the toe of each mouse using a standard protocol, and subjected to PCR for genotyping. The PCR primers for Cre were 5′-CCCGCAGAACCTGAAGATG-3′ (sense) and 5′-GACCCGGCAAAACAGGTAG-3′ (anti-sense), and the PCR primers for Catnb+/lox (exon3) mice were 5′-AGGGTACCTGAAGCTCAGCG-3′ (sense) and 5′-CAGTGGCTGACAGCAGCTTT-3′ (anti-sense).

### Animal surgical procedures

A single cortical bone defect was made in the right femoral metaphysis of all mice. The mice were anesthetized with 1% pelltobarbitalum natricum at a dose of 50 mg/kg. The right hind limb fur was shaved, the skin was disinfected, and then a towel was spread in preparation for surgery. A 10 mm incision was made over the right hind limb knee joint. The muscles were bluntly separated, and the periosteum was dissected while taking care to protect the joint cavity. An electric drill with a steel drill bit (Fine Science Tools #19007–07) with a diameter of 0.6 mm was used to drill a single 2-mm cortical defect on the articular surface. The drill bit was transferred into the marrow (medullary) cavity but did not break through the contralateral cortex [20–23]. Heat lamps were used to maintain the body temperature of the mice during their recovery from anesthesia.

### Animal groups

Six-month-old and 12-month-old male mice with the Catnb^lox(ex3)^ gene background and wild-type (WT) male littermates were divided into six groups and their femoral metaphysis fractured: adult WT group (6-month-old wild-type mice injected with saline, *n* = 12), adult PTH group (6-month-old wild-type mice injected with PTH (1–34), *n* = 8), and the adult CA (Wnt/β-catenin activation) group (6-month-old Catnb^lox (ex3)^ mice injected with tamoxifen, *n* = 8); aged WT group (12-month-old wild-type mice injected with normal saline, *n* = 12), aged PTH group (12-month-old wild-type mice injected with PTH (1–34), *n* = 8), and the aged CA group (12-month-old Catnb^lox(ex3)^ mice injected with tamoxifen, n = 8). The adult and aged PTH mice were injected daily intraperitoneally (concentration 1 μg/ml, dose 80 μg/kg body weight) with PTH (1–34) (Sigma, US) before the mice were sacrificed (7 days, 14 days, and 21 days). At the same time points, the same volume of physiological saline was administered to adult and aged WT mice by intraperitoneal injection. The adult and aged CA mice were injected with TM (75 mg/kg body weight) after surgery to activate β-catenin. To observe cell proliferation, 5′-bromo-2-deoxyuridine (BrdU, Sigma, Ireland) was intraperitoneally injected (10 μL/g body weight) 1 h and 3 days before the mice were over-anesthetized.

### Tissue preparation

Mice were sacrificed using an excess of anesthetic by Using intraperitoneal injection of sodium pentobarbital 200 mg/kg on days 7, 14, and 21 after the fracture, and RNA from the fracture area was extracted. X-ray examination was performed on the right femur of each group of four mice. These femurs were then fixed overnight using formalin and micro-CT was performed the next day. The femurs were decalcified in 10% ethylenediaminetetraacetic acid. After successful decalcification, they were tissue-embedded and sectioned (thickness: 4 μm). The sections were dewaxed to water and then histologically stained and immunohistochemically stained.

### X-ray and CT scan

X-ray examination and CT examination analysis have been widely used to assess fracture healing. The right femur X-ray image was taken on days 7, 14, and 21 following the procedure using the Faxitron system (Faxitron X-ray, Wheeling, IL, USA) [24, 25]. The right femur was examined using a Viva CT 40 (Scanco Medical, Bassersdorf, Switzerland, Switzerland) according to the procedure recommended by the American Society for Bone and Mineral Research [16]. In the examination, the femur was placed in an eppendorf tube filled with ethanol. The X-ray tube potential was 45 kVp, and the voxel size was 10 μm ^3^. The obtained image was analyzed and three-dimensionally reconstructed using EVS Beam software (1400 Hounsfield unit was set to bone) [23]. The area centered on the defect center with a diameter of 0.8 mm and a depth of 0.4 mm was set as the region of interest, and the percentage of bone volume to total volume (BV/TV) was calculated.

### Hematoxylin-eosin staining

Hematoxylin-eosin (H&E) staining was performed using the previously used procedure [18]. Briefly, tissue sections were deparaffinized to water, stained with hematoxylin for 5 min, and then differentiated with ethanolic ethanol over alkaline water. Tissue sections were stained with eosin for 15 s and then dehydrated for 2 min (twice) with 95% ethanol and then with 100% alcohol for 3 min (twice). The sections were then cleared with xylene. The slides were sealed with neutral gum [23].

### Masson’s staining for collagen

The sections were dewaxed in xylene, hydrated, stained with Weigert hematoxylin for 10 min, rinsed with distilled water, stained with Masson’s limonic acid solution for 10 min, and immersed in 2% glacial acetic acid in water. The cells were differentiated in a 1% aqueous solution of phosphomolybdic acid for 5 min, stained with aniline blue for 5 min, immersed in 0.2% aqueous glacial acetic acid solution, dehydrated with 95% alcohol and anhydrous alcohol, and then cleared with xylene before sealing with neutral gum. Blue staining in the microscopic sections indicated collagen fibers.

### Real-time polymerase chain reaction (RT-PCR)

The peri-femoral tissue was removed, approximately 1 cm of the bone was removed from the center of the fracture defect, and the bone marrow was removed by centrifugation at 5000×*g* at 4 °C, and the femur was placed in a mortar containing liquid nitrogen for grinding. The ground tissue was then placed in 1 ml TRIzol reagent to extract the total RNA. The instruments used during the experiment were treated with RNase-free DNase. The expression levels of β-catenin, PTH1R, and RUNX2 (runt-related transcription factor 2) were detected by RT-PCR using the SYBR Green assay. Glyceraldehyde-3-phosphate dehydrogenase (GAPDH) was used as the control, and the expression level of each gene was averaged relative to GAPDH. The data are expressed as the ratio of expression levels [26]. The primers are presented in Table 1.

**Table 1:** Primers used for RT-PCR

Genes	Primer Forward Sequence(5′, 3′)	Primer Reverse Sequence
β-catenin	ACGGTGCCGCGCCGCTTATA	TAGCCATTGTCCACGCAGCG
RUNX2	AAGTGCGGTGCAAACTTTCT	TCTCGGTGGCTGGTAGTGAG
PTH1R	AGCGAGTGCCTCAAGTTCAT	ACAGCGTCCTTCACGAAGAT
GAPDH	GAGAAGGCTGGGGCTCATTT	CCAATATGATTCCACCCATG
OCN	AGGGCAGCGAGGTAGTGA	TGGTGTAGCCGAAAGTCC
VEGF	CGATGAAGCCCTGGAGTG	ATGATGGCGTGGTGGTGA

Shown are the details of the primers used for RT-PCR, including the forward (F) and reverse (R) sequences. *RUNX2* Runt-related transcription factor 2, *PTH1R* Parathyroid hormone 1 receptor, *GAPDH* Glyceraldehyde-3-phosphate dehydrogenase, *OCN* Osteocalcin, *VEGF* Vascular endothelial growth factor

### Immunohistochemical staining

Immunohistochemistry (IHC) was performed with reference to a previous procedure [27]. The primary antibodies used were goat anti-rabbit osteocalcin (OCN; 1:400), runt-related transcription factor 2 (RUNX2; 1:200), β-catenin (1:300), and PTH1R (1:300), and anti-mouse matrix metalloproteinase 9 (MMP9; 1:200), vascular endothelial growth factor (VEGF; 1:200), and goat anti-rat BrdU (1:200). All antibodies were purchased from Santa Cruz Biotechnology Corporation (Santa Cruz, CA, USA). The secondary biotinylated goat anti-mouse, rabbit anti-goat, and goat anti-rabbit IgGs were purchased from Boster (Wuhan, China). Positive expression cells were observed the region of interest with an Olympus microscope, and five random regions within the observation range were selected and counted. Results were identified by positive cell values and standard deviations and used for statistical analysis.

### Tartrate-resistant acid phosphatase staining

Tissue sections were deparaffinized with xylene, hydrated, placed in tartrate-resistant acid phosphatase (TRAP) staining solution at 37 °C for 30 min, and stored in the dark. Sections were then rinsed with water, stained with methyl green for 5 min, dehydrated with gradient alcohol, cleared with toluene, and then sealed with neutral gum. The chemical reagents were purchased from Chuandong Company (Chongqing, China) [28].

The slice images were observed at a magnification of 200×, and the number of TRAP-positive cells therein was counted in five random regions. The results are displayed as the mean and standard deviation, and statistical analysis was performed.

### Statistical analysis

The experimental data used were repeated ≥3 times and are expressed as the mean ± standard deviation (BV/TV, RT-PCR, Immunostaining staining and TRAP staining). Statistical analysis was performed using one-way analysis of variance and the paired *t*-test using SPSS 19.0 software. Multiple groups of comparisons were performed by one-way analysis of variance (ANOVA) and post hoc comparisons. When the *p*-value was < 0.05, it was considered statistically significant between the observation group and the control group.

## Results

### The speed of metaphyseal fracture healing in adult CA and PTH mice was faster than in adult WT mice, but this was not statistically different

**Fig. 1:**
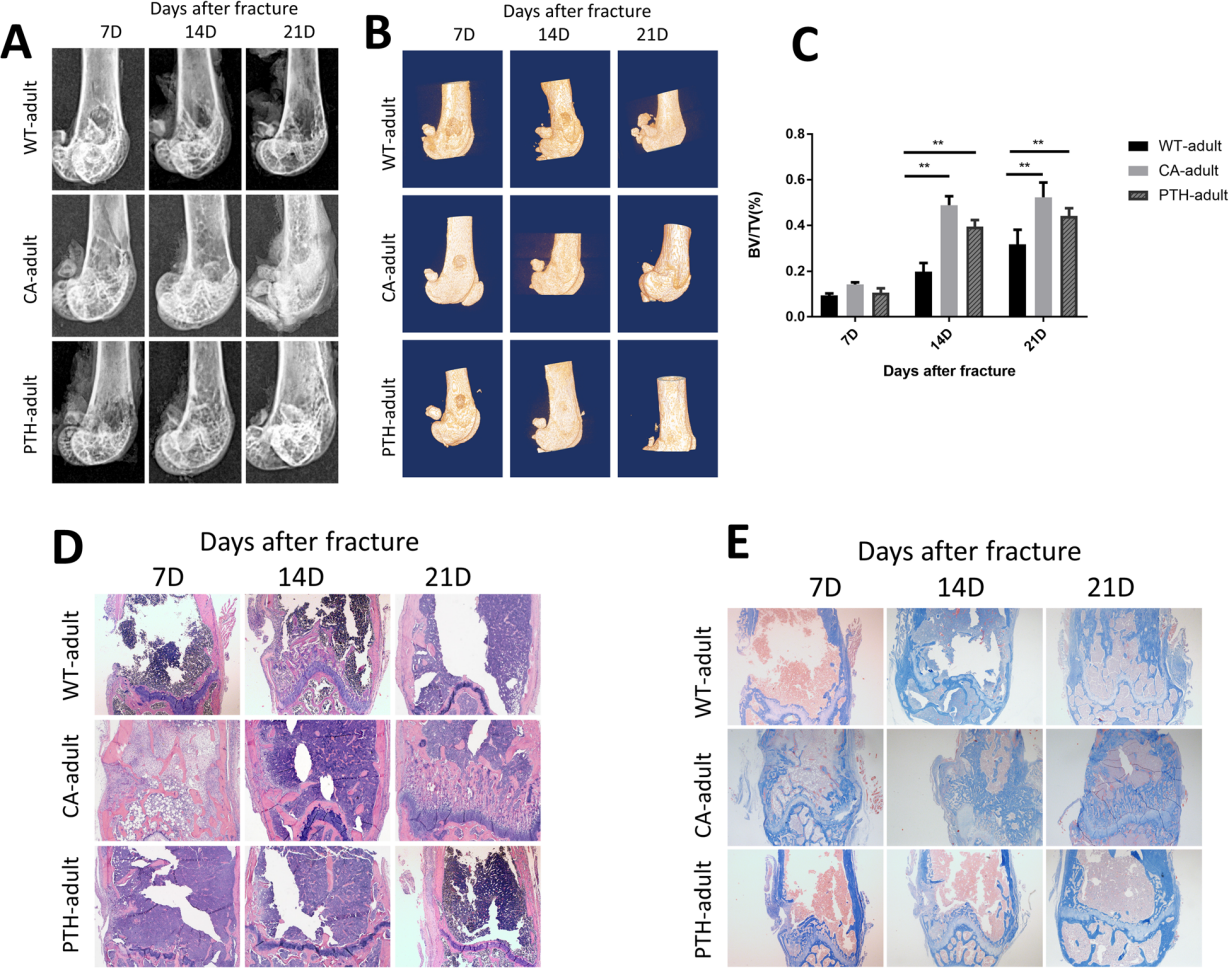
Defect healing of femoral metaphyseal fractures in adult wild-type (WT-adult) mice, adult Wnt/β-catenin activation (CA-adult) mice, and adult PTH (Parathyroid hormone) activation (PTH-adult) mice. **a** X-ray, **b** 3D-CT, **c** bone volume/tissue volume (BV/TV) within the region of interest (ROI) was calculated, **d** hematoxylin-eosin (H&E) staining of femurs (× 100), **e** Masson’s staining of the defect site of femurs (× 100). The results are expressed as the mean ± SD; *n* = 4 per group; ^*^P < 0.05 and ^**^P < 0.01

Compared with adult WT mice, we found that the metaphyseal defects in adult CA mice and adult PTH mice healed more rapidly. X-ray (Fig. 1a) examination and 3D (Fig. 1b) reconstruction images showed that on post-operative day 7, the metaphyseal bone defects of adult CA mice and adult PTH mice began to blur, but in adult WT mice the defects were still clearly observed in the middle. The extent of the metaphyseal bone defect continued to decrease on the 14th day after the fracture and disappeared 21 days after the fracture. Among the groups, it disappeared earliest in the adult CA mice and adult PTH mice.

Micro-CT quantitative examination showed that the percentage of BV/TV (Fig. 1c) in the femoral fracture defect areas of adult CA mice and adult PTH mice was much higher than in the adult WT mice at 14 and 21 days after fracture, which was the same as the X-ray examination. As with the trend of 3D reconstruction, bone defects in the metaphysis of adult CA mice and adult PTH mice healed earlier than in adult WT mice. However, no statistical difference between the fracture sites of adult CA mice and adult PTH mice was observed at the three time points by observing the percentage of BV/TV (Fig. 1c).

The same trend was observed with the H&E histological staining (Fig. 1d). On the 7th day after the fracture, soft tissue filling at the metaphyseal fracture defect of adult CA and PTH mice was observed under the microscope; in the adult WT mice, there was still a large gap and soft tissue in the metaphyseal defect. On the 14th day after surgery, more callus filling was seen at the metaphyseal defect of adult CA mice and adult PTH mice, while the metaphyseal end of adult WT mice had only soft tissue filling. On the 21st day after the fracture, healing of the metaphyseal defects in the three groups of mice was close to normal tissue.

Masson staining revealed that collagen fibers in the femoral fracture defect areas of adult CA mice and adult PTH mice were significantly more than in adult WT mice, especially on day 14 after fracture, indicating more callus (Fig. 1e).

### The speed of metaphyseal fracture healing in aged CA mice and aged PTH mice was faster than in aged WT mice, and it was faster in aged CA mice than in aged PTH mice

**Fig. 2:**
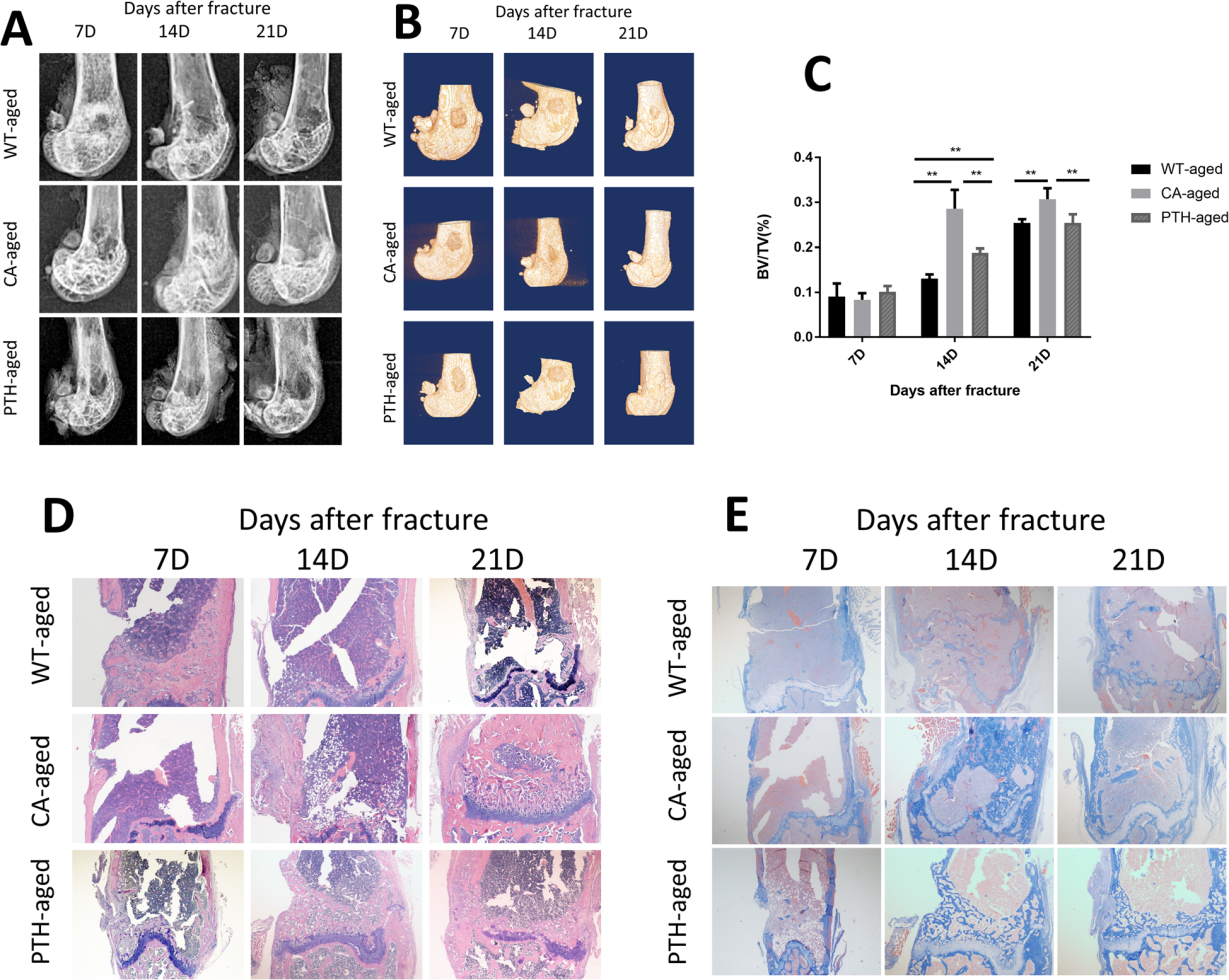
Defect healing of femoral metaphyseal fractures in elderly wild-type (WT-aged) mice, aged Wnt/β-catenin activation (CA-aged) mice, and aged PTH activation (PTH-aged) mice. **a** X-ray, **b** 3D-CT, **c** bone volume/tissue volume (BV/TV) within the ROI was calculated, **d** H&E staining of femurs (× 100), **e** Masson’s staining of the defect site of femurs (× 100). The results are expressed as the mean ± SD; n = 4 per group; ^*^P < 0.05 and ^**^P < 0.01

After activation of Wnt/β-catenin and PTH, the metaphyseal fracture defects in the aged CA mice and aged PTH mice healed faster than in the aged WT mice, as in the adult mice. X-ray (Fig. 2a) examination and 3D (Fig. 2b) reconstruction of the image suggest that on the 7th day after surgery, the area of the metaphyseal fracture defect in the aged CA mice and aged PTH mice began to become blurred, and the extent of the defect decreased further by the 14th day after surgery and was not seen on the 21st day after the fracture. In the aged WT mice, the fracture defect had not changed significantly by the 7th day after fracture, and it was clearly observed on the 14th day after the operation. The reduction was not obvious on the 21st day after the fracture. Among the mouse groups, it was also observed that the area of the fracture defect in the CA mice was smaller than in the aged PTH mice.

Micro-CT examination showed that the percentage of BV/TV in the femoral metaphyseal defect area in the aged CA mice was much higher than in the aged WT mice at 14 and 21 days after fracture, and at 14 days after fracture, the percentage of BV/TV in the defect of the femoral condyle of the aged PTH mice was higher than in aged WT mice. Interestingly, the percentage of BV/TV in the femoral metaphyseal defect area in the aged CA mice was higher than in the aged PTH mice at 14 and 21 days after fracture (Fig. 2c).

H&E staining showed that on the 7th day after the fracture, the metaphyseal fracture defects of the aged CA mice and aged PTH mice were filled with soft tissue and formed fiber connections, while the aged WT mice still had large metaphyseal defects. On the 14th day after the fracture, the fracture defect area in the aged CA mice and aged PTH mice had formed more osteophytes, while the filling tissues of the aged WT mice were still soft tissue and fibrous tissue (Fig. 2d). On the 21st day after the fracture, the healing of the metaphyseal defect in the aged CA mice was close to that of normal tissue. However, healing of the metaphyseal end in the aged PTH mice was better than in the WT mice but did not reach the healed state.

Masson staining showed that on the 7th day after the fracture, the collagen fibers in the fracture site of the aged CA mice were more than in the aged PTH mice and aged WT mice, and the trend was more obvious on the 14th and 21st days after fracture. The collagen fibers in the aged PTH mice were slightly more than the aged WT mice but did not show a significant advantage (Fig. 2e).

The percentage of BV/TV in the femoral metaphyseal defect area for all tested groups were analyzed. BV/TV were examined with a three-factor ANOVA age, treatment, and time in one omnibus analysis to compare the differences in BV/TV between the adult and aged mice in the six tested groups (Table 2). The probability level of .05 was used to assess statistical significance. There were three main effects and three two-way interactions that were statistically significant. The significant main effects can be ignored as the variables (ie, age, treatment, and time) as they are involved in the significant two-way interactions. Three two-way interactions (age*treatment, age*time and treatment*time) were found statistically significant. This analysis demonstrated that there were consistent statistically significant differences in the BV/TV in the tested groups (CA and PTH groups) as compared to the WT group at all tested time in both ages.

**Table 2:** A three-factor ANOVA test examining age, treatment and time in one omnibus analysis to compare the differences in healing between the adult and aged studied groups

Tests of between-subjects effects					
Dependent variable: BV/TV					
Source	Type III sum of squares	df	Mean square	F	Sig. *P*-value
Corrected model	1.401^a^	17	.082	82.515	.000
Intercept	4.310	1	4.310	4314.074	.000
age	.228	1	.228	227.739	.000
treatment	.186	2	.093	93.052	.000
time	.777	2	.388	388.872	.000
age * treatment	.043	2	.022	21.544	.000
age * time	.073	2	.036	36.395	.000
treatment * time	.083	4	.021	20.737	.000
age * treatment * time	.012	4	.003	3.085	.023
Error	.054	54	.001		
Total	5.765	72			
Corrected total	1.455	71			

^*a*^ *R*^2^ = .963(Adjusted *R*^2^ = .951)

### Angiogenesis and osteoblasts in the adult CA mice and adult PTH mice were greater than in the adult WT mice but without statistical difference

**Fig. 3:**
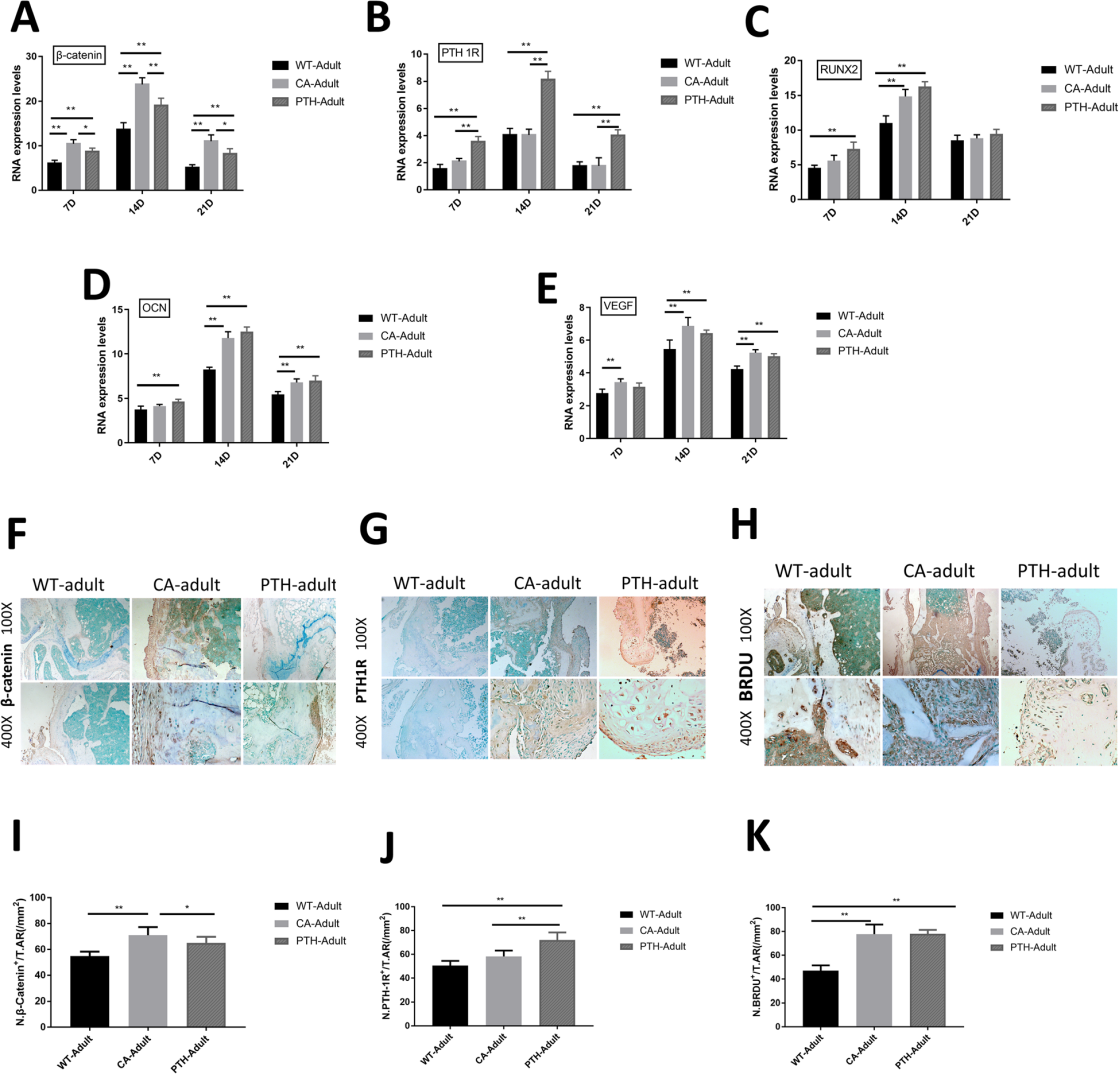
**a** β-catenin, **b** PTH1R, **c** RUNX2, **d** OCN, and **e** VEGF RT-PCR expression levels. The mRNA expression levels on the fracture day were used as an internal control. Immunostaining for (**f**) β-catenin, **g** PTH1R, and **h** BrdU at the metaphyseal defect at day 14 after fracture in adult wild-type (WT-adult) mice, adult Wnt/β-catenin activation (CA-adult) mice, and adult PTH activation (PTH-adult) mice. **i**-**k** Quantification of the number of positive cells was performed. *n* = 4 per group. ^*^P < 0.05 and ^**^P < 0.01

On days 7, 14, and 21 after fracture, the mRNA expression levels of β-catenin in the femur fractures of adult CA mice and adult PTH mice were higher than in adult WT mice, and the expression level of β-catenin was higher in adult CA mice than in adult PTH mice (Fig. 3a). On days 7, 14, and 21 after fracture, PTH1R mRNA expression levels in the femoral metaphyseal fracture region of adult PTH mice were higher than in adult CA and adult WT mice (Fig. 3b). RUNX2 mRNA expression levels in the adult PTH mouse fracture defect area were higher than in the adult WT mice on the 7th and 14th days after the fracture, and the RUNX2 mRNA was higher in the adult CA mouse than in adult WT mice on post-operative day 14. No significant differences were found between the adult CA mice and adult PTH mice at the three time points (Fig. 3c). OCN mRNA expression levels in the adult PTH mouse fracture defect area were higher than in the adult WT mice on the 7th,14th and 21th days after the fracture, and the OCN mRNA was higher in the adult CA mouse than in adult WT mice on post-operative day 14 (Fig. 3d). VEGF mRNA expression levels in the adult PTH mouse fracture defect area were higher than in the adult WT mice on the 14th and 21th days after the fracture, and the VEGF mRNA was higher in the adult CA mouse than in adult WT mice at the three time points (Fig. 3e). Immunohistochemical staining showed that the number of β-catenin-positive cells in the femoral defects of adult CA mice was greater than in the adult WT mice and adult PTH mice on post-operative day 14 (Fig. 3f and i). On day 14 post-fracture, PTH1R-positive cells in the femur defects of adult PTH mice were more abundant than PTH1R-positive cells in adult WT mice and adult CA mice (Fig. 3g and j). On the 14th day after the fracture, the number of BrdU-positive cells in the defects of adult CA mice and adult PTH mice was greater than in adult WT mice, but there was no statistical difference between adult CA mice and adult PTH mice (Fig. 3h and k).

**Fig. 4:**
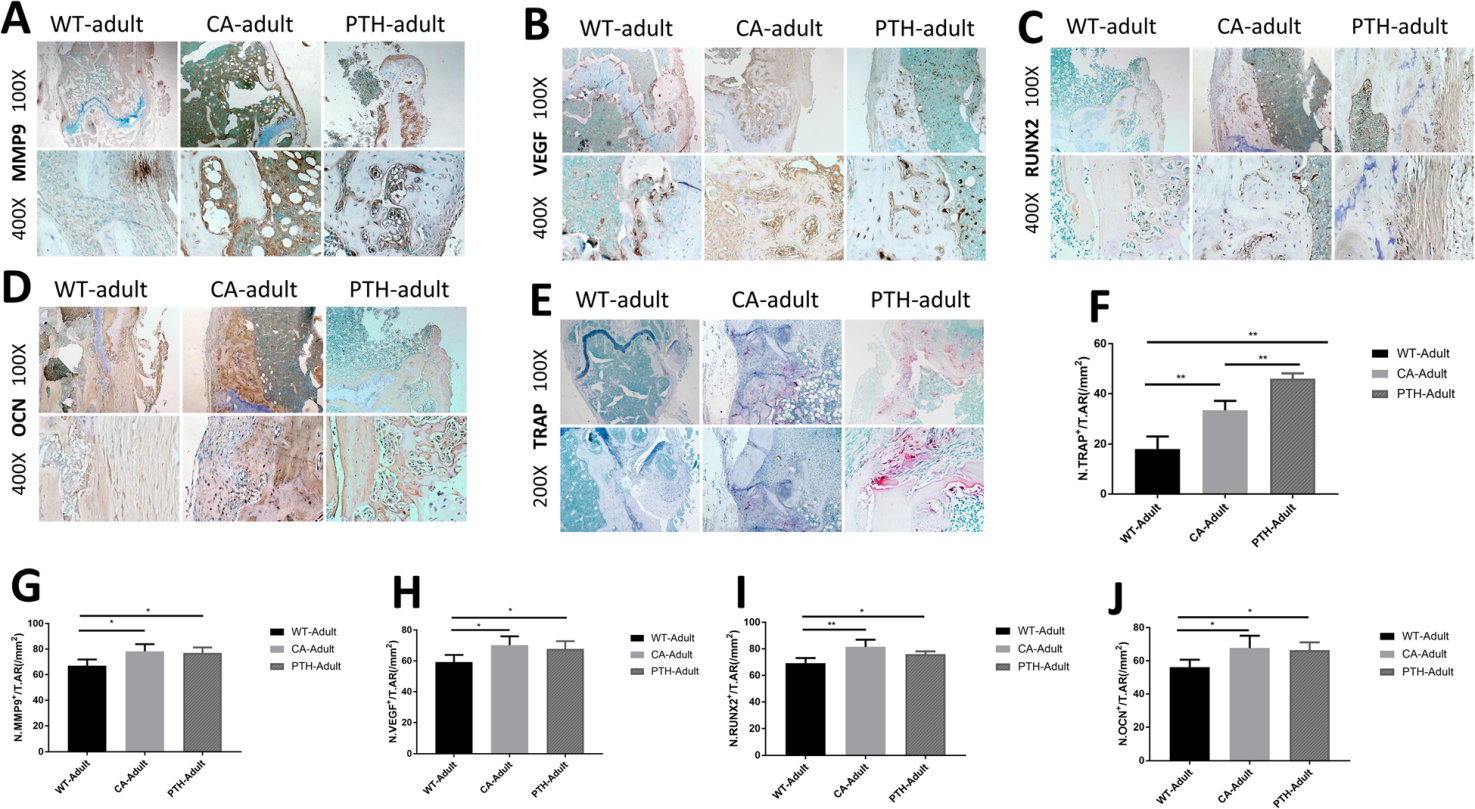
Immunostaining for **a** MMP9, **b** VEGF, **c** RUNX2, and **d** OCN, and **e** TRAP staining at the metaphyseal defect at day 14 after fracture in adult wild-type (WT-adult) mice, adult Wnt/β-catenin activation (CA-adult) mice, and adult PTH activation (PTH-adult) mice. **f** TRAP-positive cells and **g**-**j** Quantification of the number of positive cells were performed. The results are expressed as the mean ± SD; *n* = 4 per group; ^*^P < 0.05 and ^**^P < 0.01

By observing the immunohistochemical staining of the metaphysis of the femur, it was found that on day 14 after the fracture, the numbers of MMP9-positive cells (Fig. 4a and g) and VEGF-positive cells in the defects of adult CA mice and adult PTH mice (Fig. 4b and The number of H) were greater than the number in adult WT mice. Our results also found that the number of RUNX2-positive cells (Fig. 4c and i) and OCN-positive cells in the fracture defects of adult CA mice and adult PTH mice on day 14 after fracture (Fig. 4d and j) were more than that in the adult WT mice. However, there was no significant difference in the numbers of MMP9-, VEGF-, RUNX2-, and OCN-positive cells in the defects between adult CA mice and adult PTH mice, and these indexes are important for fracture healing.

On post-operative day 14, TRAP staining showed that the number of TRAP-positive cells in the femoral metaphyseal fracture of adult PTH mice was higher than in adult WT mice and adult CA mice, and the number of TRAP-positive cells in adult CA mice was higher than in adult WT mice (Fig. 4e and f).

### Angiogenesis and osteoblasts in the aged CA mice and aged PTH mice were greater than in the aged WT mice and were greater in the aged CA mice than in the aged PTH mice

**Fig. 5:**
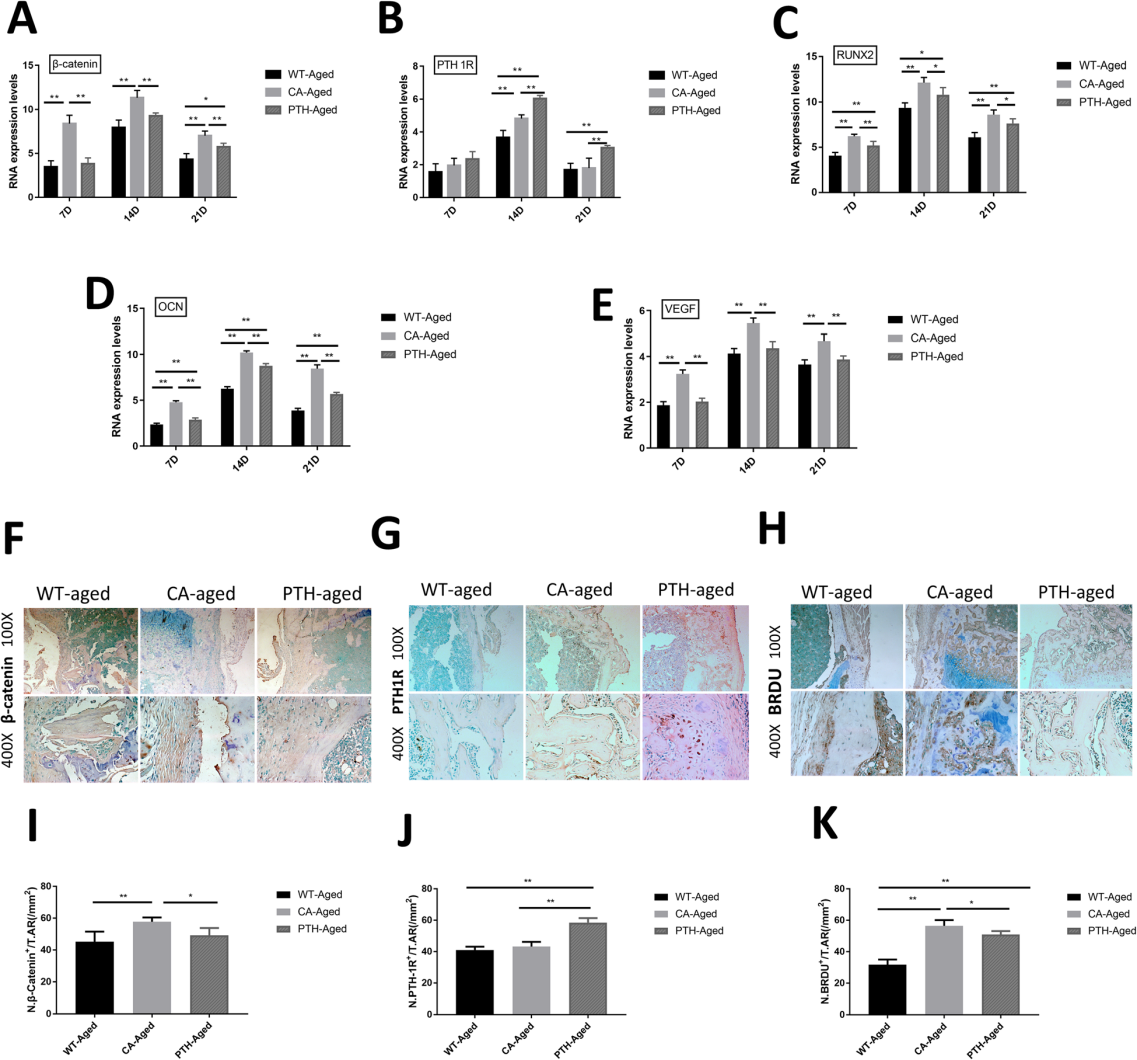
RT-PCR expression levels of **a** β-catenin, **b** PTH1R, **c** RUNX2, **d** OCN, and **e** VEGF RT-PCR expression levels and immunostaining for **f** β-catenin, **g** PTH1R, and **h** BrdU at the metaphyseal defect at day 14 after fracture in the aged wild-type (WT-aged) mice, aged Wnt/β-catenin activation (CA-aged) mice, and aged PTH activation (PTH-aged) mice. The mRNA expression levels on the day of fracture were used as an internal control. *n* = 4 per group. **i**-**k** Quantification of the numbers of positive cells was performed. ^*^P < 0.05 and ^**^P < 0.01

RT-PCR experiments showed that the expression of β-catenin mRNA in aged CA mice was higher than in aged WT mice and aged PTH mice on days 7, 14, and 21 days after fracture, and was higher in PTH mice than in WT mice on the 21st day after fracture (Fig. 5a). On day 14 and day 21 after fracture, the expression level of PTH1R mRNA in the femoral metaphyseal fracture of aged PTH mice was higher than in aged CA mice and aged WT mice, and on the 14th day after fracture the mRNA expression level of PTH1R in aged CA mice was higher than in aged WT mice (Fig. 5b). RUNX2 mRNA expression was observed in the femoral metaphyseal fracture area. On days 7 and 14 after fracture, it was higher in the aged CA and PTH mice than in the age WT mice, and was higher in the aged CA mice than in the PTH mice (Fig. 5c). OCN mRNA expression levels in the aged PTH mouse fracture defect area were higher than in the aged WT mice, and the OCN mRNA was higher in the aged CA mouse than in aged WT and PTH mice at the three time points (Fig. 3d). The VEGF mRNA was higher in the aged CA mouse than in aged WT and PTH mice at the three time points (Fig. 3e).

On post-operative day 14, immunohistochemical staining showed that the number of β-catenin-positive cells in the femoral fracture defect of the aged CA mice was higher than in the aged WT mice and aged PTH mice (Fig. 5f and i). On post-operative day 14, the number of PTH1R-positive cells in the femur fracture defect of the aged PTH mice was higher than the aged WT mice and aged CA mice (Fig. 5g and j). On post-operative day 14, the number of BrdU-positive cells in the femur fracture defect of the aged CA mice and aged PTH mice was higher than in the aged WT mice, and the number of BrdU-positive cells in the aged CA mice was higher than that in the aged PTH mice (Fig. 5h and k).

**Fig. 6:**
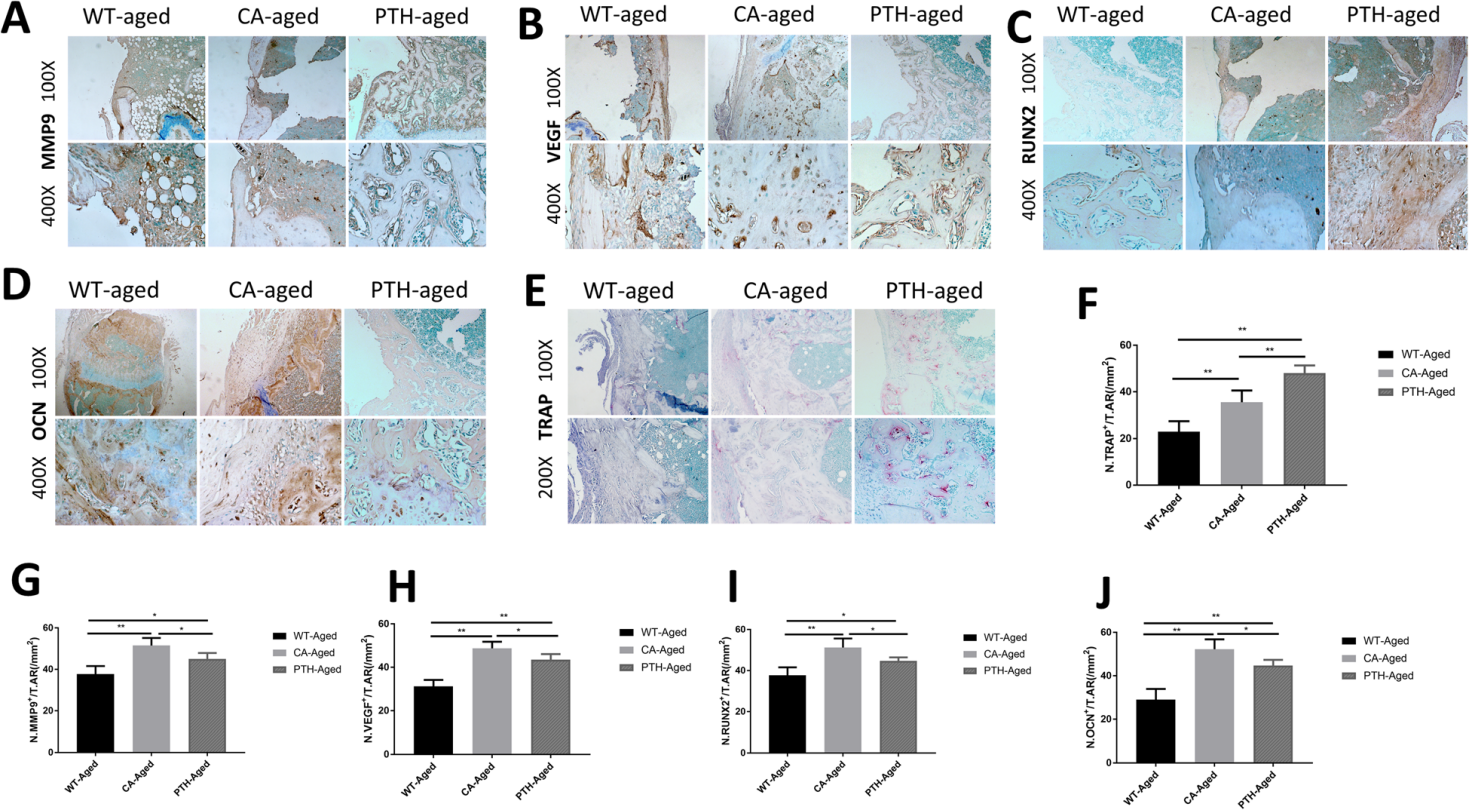
Immunostaining for **a** MMP9, **b** VEGF, **c** RUNX2, and **d** OCN, and **e** TRAP staining at the metaphyseal defect at day 14 after fracture in the aged wild-type (WT-aged) mice, aged Wnt/β-catenin activation (CA-aged) mice, and aged PTH activation (PTH-aged) mice. **f** TRAP-positive cells and **g**-**j** Quantification of the number of positive cells were performed. The results are expressed as the mean ± SD; *n* = 4 per group; ^*^P < 0.05 and ^**^P < 0.01

Development of the microcirculation in the femoral metaphyseal fracture was observed by tissue immunohistochemical staining. MMP9-positive cells were found in the femoral fracture defects of aged CA mice and aged PTH mice on the 14th day after fracture (Fig. 6a and g). The number of VEGF-positive cells (Fig. 6b and h) in the aged CA mice and aged PTH mice was greater than in the aged WT mice, and there were more in the PTH mice than in the aged CA mice. On the 14th day after the fracture, the number of RUNX2-positive cells (Fig. 6c and i) and OCN-positive cells (Fig. 6d and j) in the femur fracture defect of the aged CA mice and aged PTH mice was higher than in the aged WT mice, and the aged CA mice had more RUNX2- and OCN-positive cells than the aged PTH mice.

TRAP staining showed that the number of TRAP-positive cells in the femoral metaphyseal fracture of aged PTH mice was higher than in the aged CA mice and aged WT mice, and more in the aged CA mice than in the aged WT mice (Fig. 6e and f).

## Discussion

To form new bone and remodel, fracture healing requires a series of complex physiological changes [29]. Fractures result in a continuous middle segment of the bone and vascular damage. Fracture healing includes three main stages: inflammation, repair, and remodeling. After the fracture occurs, the fracture site forms a hematoma and releases inflammatory molecules that stimulate mesenchymal stem cells to migrate and aggregate, new microcirculation and microvessels to form, and osteoblasts to differentiate into bone cells. Finally, through deposition, remodeling forms cortical or cancellous bone [30]. It also involves hematopoiesis in the bone marrow, immune inflammatory cells, microcirculation formation, aggregation and differentiation of mesenchymal stem cells (MSC), and activation of multiple signaling molecules that regulate a cascade of events including cell migration, proliferation, differentiation, enhancement, and inhibition, as well as extracellular protein synthesis, which in turn affects osteoblast and chondrocyte differentiation [31, 32]. Microcirculation and the formation of osteoblasts and collagen fibers are important factors in the healing process of fractures.

In recent years, the Wnt/β-catenin signaling pathway and PTH signaling pathway have been the research directions for promoting fracture healing. Activation of the Wnt/β-catenin signaling pathway promotes cell proliferation, osteogenic differentiation, microcirculation, and proper inhibition of osteoblasts; angiogenesis and microcirculation are important for fracture healing [33, 34]. Many scholars have found that activation of the Wnt signaling pathway promotes microcirculation formation [35], particularly through VEGF and MMP9 [36], which have very large effects on fracture healing [37]. In our experiments, both adult and aged CA mice showed better microcirculation formation and osteogenic differentiation than the adult and aged WT mice.

In our experiments we found activation of the Wnt signaling pathway promoted the increase of VEGF mRNA expression, and the increase of VEGF and MMP9 expression in fracture defect, similar to the findings of Karki S [35, 38]. Activation of the Wnt signaling pathway promoted the increase in OCN and RUNX2 expression, thereby promoting the differentiation of osteoblasts [39–41].

Teriparatide is a recombinant human parathyroid hormone that has anabolic effects on bone and increases osteoblast activity and bone mass [42]. Because of these effects, it has recently been studied as an application to promote fracture healing. Intermittent low doses of PTH (1–34) can enhance chondrogenesis and accelerate cartilage differentiation and increase the proliferation of chondrogenic cells and type II collagen. Synthesis leads to the formation of larger cartilage calli [43, 44]. PTH can also induce cartilage callus to be converted into bone. There are also studies suggesting that PTH can modulate key molecular components of the Wnt signaling pathway during early fracture [45], promote bone morphogenetic protein signaling [46], inhibit the Notch signaling pathway [47], and aggregate mesenchymal stem cells into osteoblasts. It plays an important role in the differentiation of chondrocytes [48]. It was also found in our experiments that β-catenin was elevated during fracture healing in both adult and aged PTH mice. Adult and aged PTH mice also had more microcirculation and osteogenic differentiation than WT mice.

In our experiments, we found that activation of the Wnt/β-catenin and PTH signaling pathways in adult mice promoted the formation of osteogenesis and microcirculation and promoted cell proliferation and progression compared with WT mice. Bone differentiation promotes fracture healing. Activation of Wnt/β-catenin and PTH signaling pathways led to increased proliferation, increased expression of RUNX2 and OCN in osteoblasts, and elevated VEGF and MMP9 associated with microcirculation. Finally, morphologically, a similar promotion effect was obtained. Although Wnt/β-catenin, which inhibits osteoclasts, was activated, few TRAP cells were found in the femoral metaphyseal fracture defect than that in WT mice. The PTH mice had more osteoclasts in the fracture defect than that in WT mice and CA mice, but this did not change the morphological tendency of the adult CA and PTH mice to heal earlier. This indicates that the role of bone formation is important in the early stages of fracture healing, and the effect of osteoclasts does not lead to changes in fracture healing.

Age is a detrimental factor in fracture healing. The microenvironment of bone marrow changes with age, which may be detrimental to the proliferation of MSC or may lead to the differentiation of MSC into different cells. The differentiation of bone marrow fat increases with age, which is not conducive to osteoblast formation [49, 50]. Bergman et al. [51] found that the decrease in the number and proliferation potential of mesenchymal stem cells with age may be responsible for the decrease in the number and function of osteoblasts with age. Increased trabecular bone loss with age leads to thinning between trabecular plates, perforation and connectivity, and reduced abundance of cancellous bone, which can also adversely affect fracture healing [17].

Delayed healing in elderly patients is attributed to the lower ability of mesenchymal progenitor cells to divide and differentiate, decreased micro-angiogenic capacity, and decreased growth factor levels [52]. Some studies investigating the biomechanical advances in fractures have shown that older rats require more time to heal and restore intact mechanical strength [53–55]. Osteoporosis not only reduces bone mass but also changes the composition and structure of bone tissue [56–58]. Hormonal circulation levels change, especially the postmenopausal estrogen levels, and bone mass decreases with age. The decreased bone mass may also be due to a decrease in the anabolism of mechanical load due to decreased levels of physical activity [59].

Although the ability of fracture healing in aged mice is reduced, the corresponding molecular mechanisms and procedures still exist. In our study, the Wnt/β-catenin and PTH signaling pathways were activated to promote fracture healing, including microcirculation and osteogenesis. Activation of the Wnt/β-catenin signaling pathway and PTH signaling pathway in aged mice resulted in increased proliferation and increased expression of RUNX2 and OCN in osteoblasts, and VEGF and MMP9 associated with microcirculation compared with WT mice. Moreover, activation of the Wnt/β-catenin signaling pathway in older mice had more proliferation (BrdU expression) than with the activation of the PTH signaling pathway, increased RUNX2 and OCN expression in osteoblasts, and elevated VEGF and MMP9 associated with microcirculation. Morphologically, it was also found that activation of the Wnt/β-catenin signaling pathway in aged mice resulted in better fracture healing than activation of the PTH signaling pathway. Although activation of the PTH signaling pathway led to more osteoclasts in the femoral fracture defect than in the WT mice, the healing rate was still faster than in the WT mice. This indicates that the effect of changes in osteoclasts on the rate of fracture healing in adult mice was not as great as the effects of osteoblasts and microcirculation changes.

We considered that activation of the Wnt/β-catenin signaling pathway in older mice promoted fracture healing better than activation of the PTH signaling pathway, possibly because of the reduction in stem cells at the cancellous bone of aged mice. The microcirculation of the blood supply was reduced, and there were not enough stem cells in the fracture area to differentiate into microvessels and osteoblasts, which would not only lead to a decrease in bone density and bone quality but also a decrease in the formation of osteoblasts and microcirculation. Activation of the Wnt/β-catenin signaling pathway in the aged mice increased proliferation, osteogenic differentiation of mesenchymal stem cells, and angiogenesis. The PTH signaling pathway stimulated osteogenesis through osteoclast formation. When there were not enough stem cells in the metaphysis of the aged mice, the indirect stimulation of activation of the PTH signaling pathway did not produce more osteoblasts and microcirculation. The role of microcirculation in the healing of aged fractures is important, and the microcirculation has a considerable role in promoting the formation of osteogenesis. Activation of the Wnt/β-catenin signaling pathway in aged mice led to stronger proliferation, osteogenesis, and microcirculation formation, which promoted the early fracture healing better than with the activation of the PTH signaling pathway.

During fracture healing, the relevant cellular molecules from the soft tissue, endothelium, and blood vessels need to reach the fracture site, in which the trabecular bone in the cancellous bone is surrounded by the bone marrow. The stem cells are relatively easy to obtain during healing and differentiate into osteoblasts and microvessels. In adult mice, there are sufficient microcirculation and stem cells in the cancellous bone for transformation, irrespective of whether the Wnt/β-catenin signaling pathway is activated to directly promote osteogenic differentiation and microcirculation or the PTH signaling pathway is activated to promote osteogenic differentiation indirectly. And, it does not cause too much difference in promoting fracture healing.

## Conclusion

Although activation of the Wnt/β-catenin signaling pathway in adulthood shows some advantages over activation of the PTH signaling pathway, there was no statistically significant difference; in the older animal, it was significantly better than the activation of the PTH signaling pathway. This may prompt us to consider that using drugs related to the Wnt signaling pathway may be better in the early stages of fracture healing in the elderly.

## Data Availability

The datasets used and/or analyzed during the current study are available from the corresponding author on reasonable request.

## Abbreviations

BrdU: 5′-bromo-2-deoxyuridine
BV/TV: Bone volume to total volume
GAPDH: Glyceraldehyde-3-phosphate dehydrogenase
H&E: Hematoxylin-eosin
IHC: Immunohistochemistry
MMP9: Matrix metallopeptidase 9
OCN: Osteocalcin
RT-PCR: Real-time polymerase chain reaction
RUNX2: Runt-related transcription factor 2
TRAP: Tartrate-resistant acid phosphatase
VEGF: Vascular endothelial growth factor
WT: Wild-type
